# The Na^+^/H^+^ Exchanger Controls Deoxycholic Acid-Induced Apoptosis by a H^+^-Activated, Na^+^-Dependent Ionic Shift in Esophageal Cells

**DOI:** 10.1371/journal.pone.0023835

**Published:** 2011-08-22

**Authors:** Aaron Goldman, HwuDauRw Chen, Mohammad R. Khan, Heather Roesly, Kimberly A. Hill, Mohammad Shahidullah, Amritlal Mandal, Nicholas A. Delamere, Katerina Dvorak

**Affiliations:** 1 Department of Cellular and Molecular Medicine, University of Arizona, Tucson, Arizona, United States of America; 2 Department of Physiology, University of Arizona, Tucson, Arizona, United States of America; 3 Arizona Cancer Center, University of Arizona, Tucson, Arizona, United States of America; Purdue University, United States of America

## Abstract

Apoptosis resistance is a hallmark of cancer cells. Typically, bile acids induce apoptosis. However during gastrointestinal (GI) tumorigenesis the cancer cells develop resistance to bile acid-induced cell death. To understand how bile acids induce apoptosis resistance we first need to identify the molecular pathways that initiate apoptosis in response to bile acid exposure. In this study we examined the mechanism of deoxycholic acid (DCA)-induced apoptosis, specifically the role of Na^+^/H^+^ exchanger (NHE) and Na^+^ influx in esophageal cells. In vitro studies revealed that the exposure of esophageal cells (JH-EsoAd1, CP-A) to DCA (0.2 mM -0.5 mM) caused lysosomal membrane perturbation and transient cytoplasmic acidification. Fluorescence microscopy in conjunction with atomic absorption spectrophotometry demonstrated that this effect on lysosomes correlated with influx of Na^+^, subsequent loss of intracellular K^+^, an increase of Ca^2+^ and apoptosis. However, ethylisopropyl-amiloride (EIPA), a selective inhibitor of NHE, prevented Na^+^, K^+^ and Ca^2+^ changes and caspase 3/7 activation induced by DCA. Ouabain and amphotericin B, two drugs that increase intracellular Na^+^ levels, induced similar changes as DCA (ion imbalance, caspase3/7 activation). On the contrary, DCA-induced cell death was inhibited by medium with low a Na^+^ concentrations. In the same experiments, we exposed rat ileum *ex-vivo* to DCA with or without EIPA. Severe tissue damage and caspase-3 activation was observed after DCA treatment, but EIPA almost fully prevented this response. In summary, NHE-mediated Na^+^ influx is a critical step leading to DCA-induced apoptosis. Cells tolerate acidification but evade DCA-induced apoptosis if NHE is inhibited. Our data suggests that suppression of NHE by endogenous or exogenous inhibitors may lead to apoptosis resistance during GI tumorigenesis.

## Introduction

Esophageal adenocarcinoma (EAC) is one of the most aggressive malignancies with an low five-year survival rate [1]. In the last three decades EAC incidence increased by more than 600% [2]. EAC now has the fastest growing incidence rate of all cancers in the U. S. [2].

The major risk factor for the development of EAC is gastroesophageal reflux disease (GERD) [3]. The esophageal epithelium is exposed to acid and hydrophobic bile acids during reflux episodes. There is evidence suggesting that the concentrations of bile acids are increased in the refluxate of patients with Barrett's esophagus (BE) and are even higher in patients with esophageal adenocarcinoma (EAC) [3]. Hydrophobic bile acids, such as deoxycholic acid (DCA), induce apoptosis [4], [5]. However, chronic, long-term exposure of cells to bile acids leads to the selection of clones that are unable to activate apoptosis [6]. Resistance to bile acid-induced apoptosis is one of the characteristics of gastrointestinal malignancies including esophageal adenocarcinoma [7]. To identify how the cells avoid apoptosis in response to bile acids, we first need to understand the molecular changes that are activated to eliminate damaged cells after bile acid exposure.

The various different mechanisms that have been suggested to contribute to bile acid-induced apoptosis include oxidative stress, mitochondrial damage, ER damage, or the activation of cell death receptors, Fas and TRAIL-R2 [8], [9], [10], [11], [12], [13], [14], [15]. Interestingly, one study in colon cancer cells showed that deoxycholic acid (DCA)-induced apoptosis is associated with altered cytoplasmic ion concentrations [16]. However, no specific mechanism was determined in this study.

The ubiquitously expressed sodium hydrogen exchanger (NHE) has a major influence on cytoplasmic ion concentrations and cell volume [17]. There are 9-subfamily NHE members in the solute carrier family 9 (SLC9) gene family. These exchangers move Na^+^ into the cell and H^+^ out of the cell to regulate cell volume, pH and ion homeostasis [17], [18]. During cellular stresses, such as hypoxia or oxidative stress, inhibition of the NHE function may prevent apoptosis [19], [20] and endogenous molecules such as nitric oxide (NO) may partially suppress NHE activity [21], [22].

In this study we examined the role of NHE and ion imbalance in DCA-induced apoptosis in esophageal cells. We hypothesized that the initial acidification induced by DCA is associated with perturbation of acidic lysosomes. To normalize intracellular pH, cells attempt to extrude acid through the NHE. This is accompanied by Na^+^ influx, loss of K^+^ and increase of cytoplasmic Ca^2^. This chain of events may control the activation of programmed cell death following DCA treatment [23], [24], [25].

## Methods

### Ethics Statement

All animal protocols were approved by the Animal Care and Use Committee of the University of Arizona; PHS Assurance number A-324801-95081 and protocol number 07-093. Three week old Sprague–Dawley rats (Charles River Labs, Pontage, MI) were terminated via anaesthetization followed by decapitation prior to any experimental or surgical work. These methods have been approved for the use of animals for scientific exploration by national and international guidelines.

### Cell lines and chemicals

JH-EsoAd1 esophageal adenocarcinoma cells were a kind gift from Dr. James R. Eshleman (Johns Hopkins University, Baltimore, MD) [26]. The cells were cultured in RPMI containing 10% FBS. CP-A cells (derived from patient with nondysplastic BE) were kindly provided by Dr. Rabinovitch (Fred Hutchinson Cancer Research Center, University of Washington). The cells were maintained in MCDB 153 medium as described previously [27]. All experiments were performed in a serum-free, phenol red-free RPMI medium (Sigma, St. Louis MO). Deoxycholic acid (DCA) was purchased from MP Biochemicals (Cleveland, OH). Amphotericin B (AMB) was used at a concentration of 50 ng/ml (Invitrogen, Carlsbad, CA), Ethylisopropyl-amiloride (EIPA) and ouabain were diluted in dimethylsulfoxide (DMSO) to 50 mM and used at a final concentration of 20 uM and 100 uM, respectively. Dimethylamiloride (DMA), Go6983 and zoniporide were obtained from Sigma, Calbiochem (San Diego, CA), and Tocris Bioscience (Ellisville, MI), and were used at final concentration of 100 uM, 10 uM and 20 uM, respectively. The composition of Krebs solution was: NaCl 117 mM, KCl 4.5 mM , CaCl_2_ 1.5 mM, MgCl_2_ 1.0 mM, NaHCO_3_ 20 mM, glucose 6.0 mM, HEPES 10 mM. NaCl was substituted with choline chloride to prepare solution with reduced Na^+^. Probes for pH measurement, 2′-7′-bis(carboxyethyl)-5(6)-carboxyfluorescein-AM (BCECF-AM); calcium, Fura-2-AM; lysosomes; Lysotracker-Green/Red; nuclear stains, DAPI and Hoechst 33342, were obtained from Molecular Probes (Invitrogen, Carlsbad, CA).

### Measurement of intracellular sodium and potassium

Sodium and potassium were measured by atomic absorption spectrophotometry following a published method [28]. Briefly, cells grown to confluence on 6-well culture dishes were pre-incubated in serum-free RPMI for 30 minutes with or without EIPA (pH 7.4) at 37°C. After incubation with treatment medium +/− DCA at varying concentrations for 60 min at 37°C, media was removed and washed two times with ice-cold isotonic magnesium chloride solution (100 mM MgCl_2_ in nano-pure, double distilled H_2_0). The magnesium chloride solution was removed and cells were digested in 30% nitric acid over night at room temperature under steady rocking. The acid digest was diluted with nano-pure, double distilled H_2_0 and the sodium or potassium content of the diluted cell lysate was measured using an atomic absorption spectrophotometer (Analyst 100; Perkin-Elmer, Norwalk, CT) at wavelengths of 589.0 or 766.5 nm, respectively. An aliquot of the acid digest was neutralized with 5 M NaOH and applied to a BCA assay (Thermo Scientific, Rockford, IL) to determine total protein per treatment well.

### Intracellular pH measurement

Intracellular pH (pH_i_) was measured in cells by epifluorescence microscopy using the pH-sensitive dye BCECF as described previously but under non-perfused conditions [21]. Six individual pH_i_ measurements from at least 25 cells were taken at each time point. These values were averaged to determine the pH_i_ at: 0 minutes (immediately prior to treatment), 5, 20, 40, 60 minutes following the indicated treatments. pH_i_ values were subtracted from pH_i_ values obtained from a control cell line incubated in normal medium and evaluated at each time point. Values are expressed as the change in pH_i_ from control at the respective time point. These experiments were performed on at least 3 independent occasions totaling ∼75 cells for each time point.

### Measurement of intracellular calcium

Cytoplasmic calcium was determined in cells loaded with the calcium-sensitive dye Fura-2-AM, dissolved in 20% pluronic F-127 in DMSO from Molecular Probes (Invitrogen, Carlsbad, CA) by a method described previously [29]. Briefly, cells were grown to semi-confluence on 35 mm glass bottom culture dishes (MatTek Corp, Ashland, MA). Cells were then loaded with 3 uM Fura-2 AM for 30 minutes under humidified 5% CO_2_ at 37°C. The cells were then washed three times with serum-free, phenol red-free RPMI solution and placed back at to de-esterify for an additional 15 minutes and then incubated in treatment conditions for 60 minutes at 5% CO_2_ and 37°C. Fura-2 fluorescence intensity was measured using an emission wavelength of 520 nm and alternating dual excitation wavelengths of 340 nm and 380 nm read by a digital imaging system (InCyt Im2; Intracellular Imaging Inc., Cincinnati OH).

### Contrast Microscopy

Phase contrast images of live cells were captured using a 20× objective with a 1.5× optivar lens on an Olympus IMT-2 using a Hamamatsu Orca 100 camera and SimplePCI ver 6.2 software (Hamamatsu, Tokyo, Japan).

### Immunofluorescence and visualization/quantification of lysosomes

Immunofluorescence was performed by a method described previously [27] using a mouse monoclonal antibody against lysosome-associated protein 1 (LAMP-1,1∶100 Santa Cruz Antibodies, Santa Cruz, CA) in conjunction with confocal microscopy.

Lysotracker-Red fluorescent probe was incubated with formalin fixed cells, following indicated treatments, for 15 minutes at a working concentration of 200 nM. DAPI was used as a nuclear counterstain. Quantification of lysosomes was performed in white 96-well plates (Corning inc, Corning, NY). Cells were grown to confluence and exposed to the indicated treatments. Following exposure to treatment cells were washed 2 times and incubated with 200 nM Lysotracker-Green and Hoechst 33342 at 0.5 mg/ml for 15 minutes. Then the cells were washed two times and read with ex/em 355/460 nm to detect the nuclear stain and then read at ex/em 485/535 nm to detect Lysotracker Green using the Victor3V multi-label fluorescent plate reader (Perkin Elmer, Waltham, Massachusetts, USA). Values were calculated as f_lysotracker_/f_Hoechst_ and the data are expressed as % of control.

### RT real-time PCR

Total RNA was isolated from cells using the RNeasy Plus Mini Kit (Qiagen, Santa Clarita, CA) as described in the manufacturer's protocol. RNA concentration and purity was evaluated by NanoDrop (ThermoScientific, Wilmington, DE) at 260 nm/280 nm. RT real-time PCR assays were performed to quantify mRNA levels of SLC9A1, SLC9A2 and SLC9A3 as described previously [30]. Primers were obtained from Real Time Primers, LLC (Elkins Park, PA). The following sequences were used for each of the primers: SLC9A1 : forward primer 5′- CCC AGG ATT GTG CAA TAG TC -3′; reverse primer 5′- GTA CGT GGT TGT CGA TGT CA -3′; SCL9A2: forward primer 5′- AAG GAT GCA AGT GCC TAC AG -3′ reverse primer 5′- TGC TCA ACA ATC CAG CAG TA -3′; SCL9A3: forward primer 5′- GAG TCC TTC AAG TCG ACC AA -3′ reverse primer 5′- CCT TGA TGG TGA AAT TCT GC -3′, b-actin: forward primer 5′- AGA-GGG-AAA-TCG-TGC-GTC-AC-3′ reverse primer 5′- CAA-TAG-TGA-CCT-GGC-CGT-3′).

### Electron microscopy

Transmission electron microscopy was used to detect morphological differences between control cells and cells treated for 60 min with control medium or DCA in the presence or absence of EIPA. Following treatments, the cells were immediately fixed with 3% glutaraldehyde in 0.1 mM cacodylate buffer (pH 7.2). The cells were postfixed in 1% osmium tetroxide, dehydrated in a graded series of ethanols, and embedded in epoxy resin. Ultrathin sections were evaluated for morphological changes using a Phillips CM12 transmission electron microscope (Eidenhoven, Netherlands).

### Cell death/cytotoxicity and caspase 3/7 activity measurement

Cells were treated for 2 hours with treatment medium and recovered in control medium for 24 hours. Using brightfield microscopy two hundred cells were evaluated (1,000×) for apoptosis and necrosis as described previously [31] [27]. MTS assay was used to evaluate the cytotoxic effect and measure cell viability 24-hours after treatment as described previously [27] [21].

Cells were grown to semi-confluence on 96-well clear (Franklin lakes, NJ) and black plates (Nunc, Roskild, Denmark). Twenty-four hours following a 2 hour treatment in the indicated conditions the Apo-ONE® caspase assay (Promega, Madison, WI) was performed following manufacturer protocol using the Victor3V multi-label fluorescent plate reader at ex/em 485/535 nm (Perkin Elmer, Norwalk, CT). Con-current MTS assay with treatments performed simultaneously was used to normalize caspase activity between treatments. Values were calculated as f_Apo1caspase_/cell viability and the data are expressed as % of control.

### ATP measurement

Cells were exposed for 2 hours to various concentrations of DCA in the presence or absence of EIPA. Then, ATP levels were measured by Enliten ATP Assay System Bioluminiscence Kit (Promega) according the manufacturer's instructions.

### Ex-vivo culture

Three week old Sprague–Dawley rats were terminated via anaesthetization followed by decapitation. The ileum was removed and flushed with RPMI media containing 1% penicillin/streptomycin. The ileum was then cut into 5 mm segments followed by a longitudinal cut to expose the villi. Tissue was immediately immersed in RPMI +/− 20 uM EIPA for 30 minutes at room temperature and then exposed to the indicated treatment medium for 2 hours. Tissue was used for immunoblotting and immunohistochemistry as described below.

### Immunoblotting

Ileum samples were homogenized in freshly prepared cell lysis buffer [27] in the presence of 1× HALT protease/phosphatase inhibitor (Fisher Scientific, Rockford, IL). The lysate was subjected to immunoblotting as described previously [32]. Anti-active caspase-3 antibody mouse monoclonal (Abcam, Cambridge, MA) was used at dilution 1∶500. β-actin (1∶5,000) antibody was used as a loading control (Oncogene Research Products, Cambridge MA).

### Immunohistochemistry

Immunohistochemistry was performed as previously described [33] using antibody against cleaved caspase-3 (1∶100, Cell Signaling, Boston, MA).

### Statistics

ANOVA was used to identify differences between individual groups and multiple groups. The data are expressed as mean ± SEM.

## Results

### DCA induces an increase of intracellular Na^+^ and activation of the Na^+^/H^+^ exchanger (NHE)

**Figure 1:**
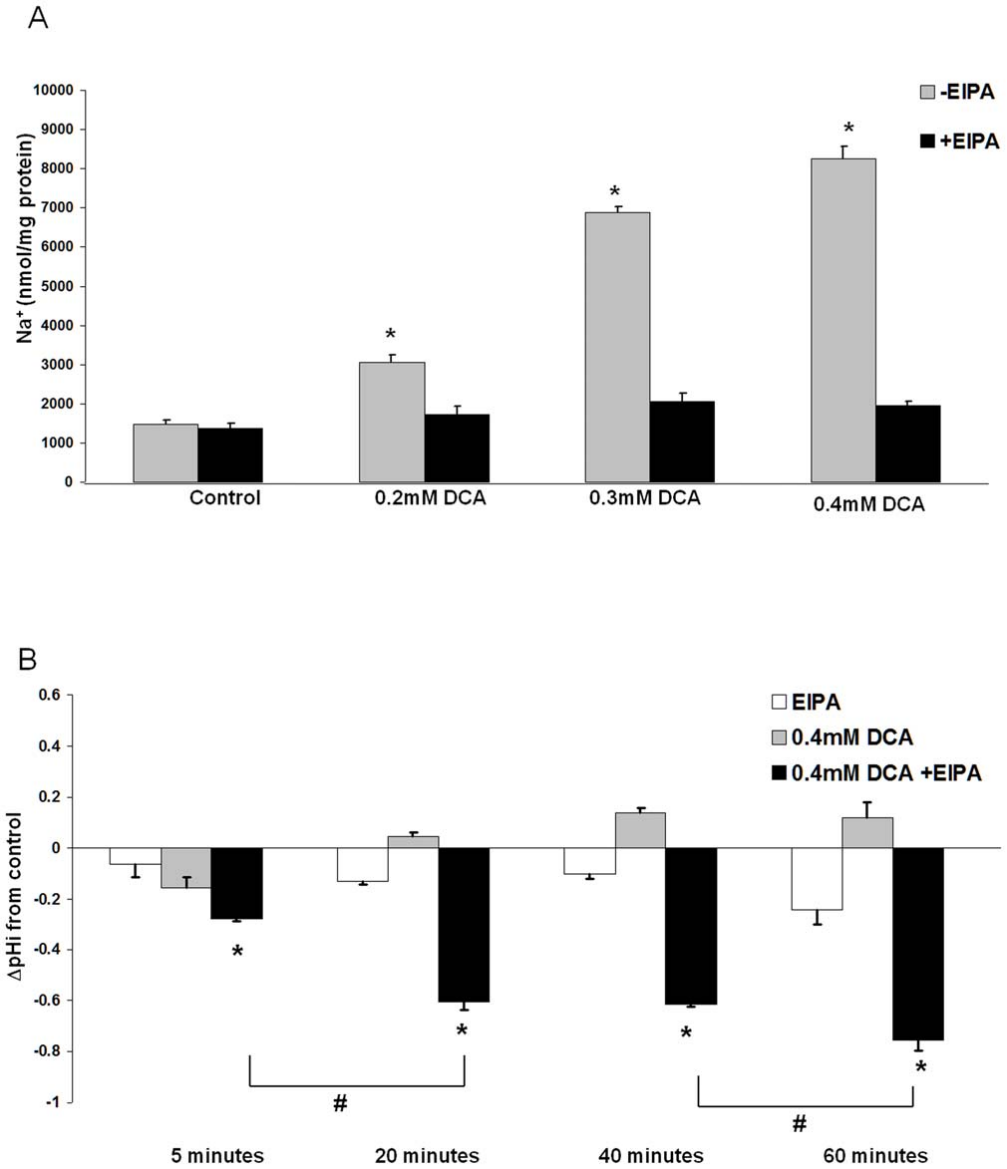
Na^+^/H^+^ exchanger (NHE) is responsible for DCA-mediated Na^+^ influx. A) Total cellular Na^+^ levels in JH-EsoAd1 cells (n = 6) after 60 minute incubation with various concentrations of DCA in the presence or absence of 20 uM EIPA. Values were calculated as nmol Na^+^/mg of protein (*p<0.01 compared to untreated cells). B) Graph shows intracellular pH (pH_i_) measured in JH-EsoAd1 cells at 5, 20, 40 and 60 minute during indicated treatments. Data are expressed as change in pH_i_ from control (*p<0.01 compared to DCA alone or as indicated #p<0.01).

EAC cells (JH-EsoAd1) were treated for 60 minutes with concentrations of DCA in the range 0.2 mM to 0.4 mM which are concentrations reported in patients with Barrett' esophagus [34]. Total cellular levels of Na^+^ increased in a dose-dependent manner (Figure 1A, p<0.001). Several mechanisms may contribute to the increased intracellular Na^+^ observed during exposure to DCA. Since our previous studies suggested that bile acids induce NHE-dependent intracellular acidification, we hypothesized that NHE may be implicated in the alteration of intracellular H^+^ and Na^+^ levels [21]. Here we show that the selective NHE inhibitor, ethylisopropyl-amiloride (EIPA, 20 uM), [35], completely prevented the increase of Na^+^ in response to DCA (Figure 1A).

We reasoned that if NHE activation is responsible for Na^+^ influx, the intracellular pH (pH_i_) of DCA-treated cells will be different in the presence or absence of EIPA. Indeed, we observed a transient acidification that occurred following addition of DCA at 5 minutes (Figure 1B, gray bars). Over time, however, there was a trend toward alkalinization consistent with H^+^ export by NHE (Figure 1B, gray bars). Inhibition of NHE with EIPA caused a large fall of cytoplasmic pH in the cells exposed to DCA (Figure 1B, black bars). These results are consistent with the notion that DCA-induced decrease of pH_i_ is responsible for activation of NHE and subsequent increase of intracellular Na^+^ (Figure 1A).

### DCA-mediated lysosomal membrane perturbation correlates with intracellular acidification

**Figure 2:**
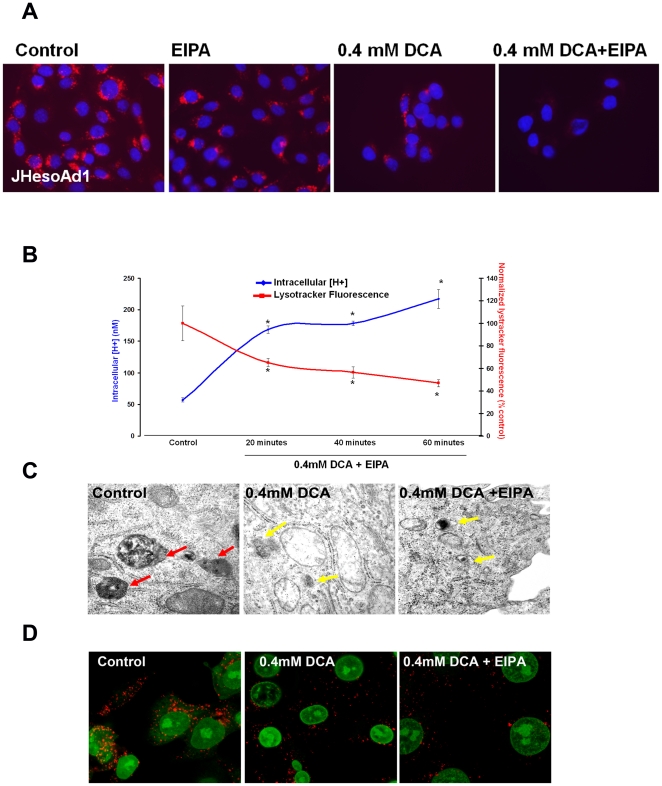
Lysosomal damage accompanies DCA-mediated intracellular acidification. A) Representative images from fluorescent microscopy of Lysotracker-Red in JH-EsoAd1 cells following 60 minutes incubation with or without 0.4 mM DCA in the presence or absence of 20 uM EIPA. Red signal indicates acidic vesicles, blue signal is nuclear counterstain (DAPI). B) Intracellular [H^+^] and quantification of Lysotracker-Green fluorescence (ex/em 485/535 nm) normalized with Hoechst 33342 nuclear stain (ex/em 355/460 nm) in cells (n = 12 for each condition) following 60 minutes with or without 0.4 mM DCA in the presence of 20 uM EIPA. Data are expressed as % of control (*p<0.05). C) Representative image (31,000×) of cells incubated for 60 minutes with or without 0.4 mM DCA in the presence or absence of 20 uM EIPA. Red arrows indicate structurally intact lysosomes, yellow arrows indicate lysosomal membrane perturbation. D) Representative confocal microscopy images showing LAMP-1 (green signal) and nuclear stain for propidium iodide (red signal) in cells incubated for 60 minutes with or without 0.4 mM DCA in the presence or absence of 20 uM EIPA.

To examine the mechanism responsible for the observed intracellular pH decrease after DCA exposure we analyzed lysosomes using the fluorescent dyes, LysoTracker-Green and LysoTracker Red [36]. LysoTracker Red staining showed a punctate pattern consistent with lysosomal distribution. DCA reduced the intensity of Lysotracker-Red within the cell. This effect was not prevented by addition of EIPA (Figure 2A). The observed change in the intensity of Lysotracker fluorescence is consistent with lysosomal membrane permeabilization [37], [38]. Lysosomal perturbation and intracellular pH reduction occurred simultaneously (Figure 2B).

Electron microscopy was used to visualize individual lysosomes. Intact lysosomes (red arrows, Figure 2C) were evident in control cells. However, cells treated with DCA showed lysosomes which appeared to leak contents into the cytosol. This effect of DCA on the lysosomes was not prevented by EIPA (Figure 2C). We reasoned that if bile acids cause lysosomal breakdown the expression of lysosomal-associated membrane protein 1 (LAMP1) would diminish. This was the case. Loss of LAMP1, compared to control, confirmed that DCA, in the presence or absence of EIPA, induces perturbation of lysosomal membrane integrity and dissemination of lysosomal contents (Figure 2D). Since this is an important finding we used another esophageal cell line to confirm that the effect of DCA is not unique to JH-EsoAd1 cells. The similar results were obtained in cells derived from Barrett's esophagus (CP-A cells, Figure S1).

### DCA-induced Na^+^-influx is required for changes of intracellular K^+^ and Ca^2+^

**Figure 3:**
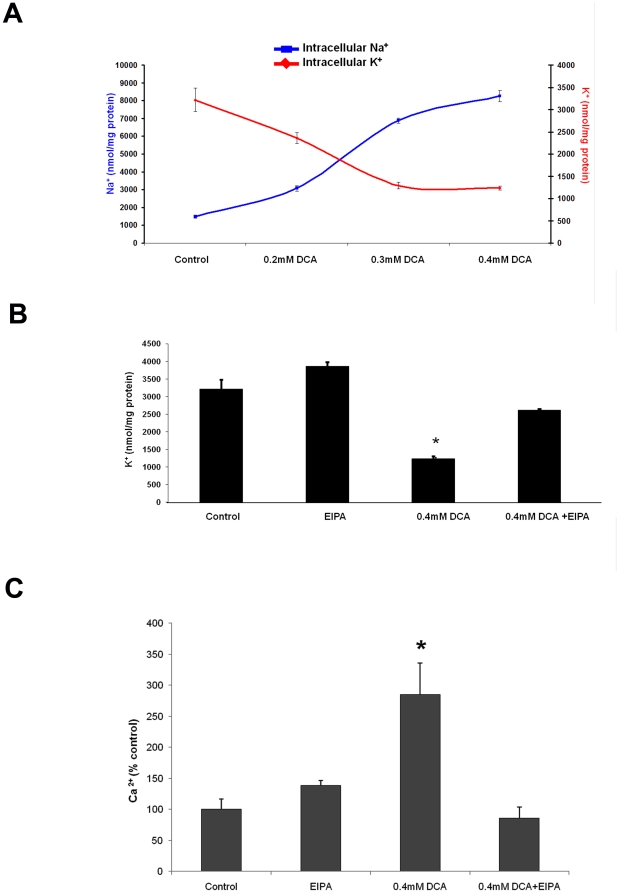
Inhibition of NHE prevents DCA-induced K^+^ efflux and rise of cytoplasmic Ca^2+^. A) Total cell K^+^ (red trace) and Na+ (blue trace) levels in JH-EsoAd1 cells following incubation for 60 minutes with or without 0.4 mM DCA. The data are expressed as nmol ion/mg protein (n = 6). B) Total cell K^+^ levels in JH-EsoAd1 cells (n = 6) following 60 minute incubation with or without 0.4 mM DCA in the presence or absence of 20 uM EIPA (*p<0.05 compared to control). C) Cytoplasmic Ca^2+^ in JH-EsoAd1 cells (n≥20 cells for each condition from at least 2 independent experiments) determined by Fura-2 fluorescence following a 60 minute incubation ±0.4 mM DCA in the presence or absence of 20 uM EIPA, (*p<0.05 compared to control).

Na^+^ influx typically results in the changes of intracellular concentrations of K^+^ to preserve osmotic equilibrium [24]. We analyzed K^+^ levels in the cells exposed to DCA for 60 minutes and observed a decline of intracellular K^+^ which paralleled the Na^+^ rise in a dose-dependent manner (Figure 3A). Importantly, suppression of NHE activity with EIPA, reduced the magnitude of DCA-induced K^+^ decrease (Figure 3B p<0.01).

In cells that have an abnormally high cytoplasmic Na^+^ concentration, Ca^2+^ export via Na^+^-Ca^2+^ exchange fails to occur [39].. We measured cytosolic Ca^2+^ using the calcium-sensitive dye, Fura-2. Intracellular Ca^2+^ levels increased significantly following 60 minute incubation with DCA. Ca^2+^ rose by more than 285%±25% compared to untreated cells (Figure 3C). Importantly, the increase in cytosolic [Ca^2+^] was abolished by EIPA (Figure 3C).

### DCA-induced apoptosis requires NHE-mediated Na^+^-influx

Our studies show that DCA exposure leads to an increase in intracellular Na^+^ levels, which results in a decrease of K^+^ and a rise in Ca^2+^. The ion changes appear to be linked to NHE activation because they are prevented by EIPA. We tested whether NHE activation influences apoptosis in DCA-treated cells.

**Figure 4:**
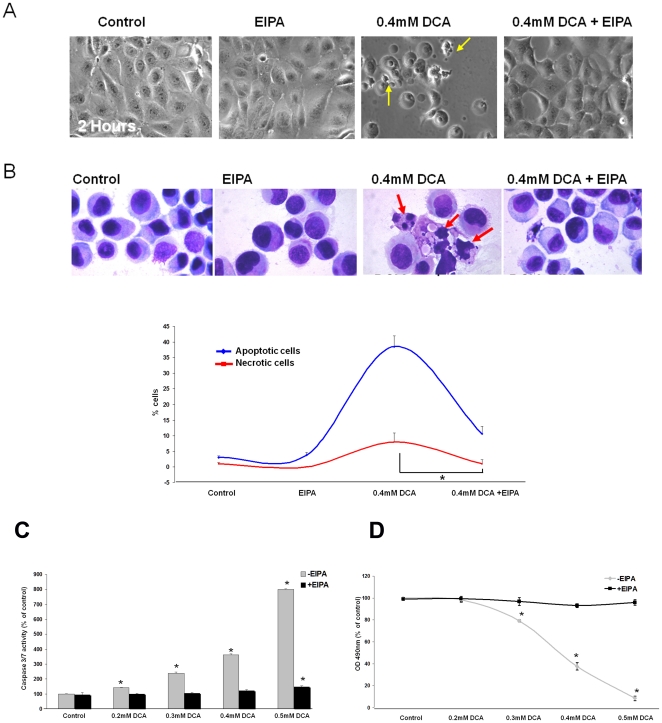
Inhibition of Na^+^ influx with EIPA prevents DCA-induced cell death. A) Representative contrast microscopy images of JH-EsoAd1 cells following 120 minute incubation with and without 0.4 mM DCA in the presence or absence of 20 uM EIPA. Yellow arrows indicate damaged and apoptotic cells. B) The upper panel shows representative images of methanol-fixed cells stained with Geimsa (red arrows indicate apoptotic and damaged cells). The lower panel shows quantification of Geimsa stained cells (n = 6 for each condition) analyzed 24 hours after a 120 minute incubation with or without 0.4 mM DCA in the presence or absence of 20 uM EIPA. Data are expressed as % of apoptotic or necrotic cells (red trace indicates necrotic cells, blue trace indicates apoptotic cells,*p<0.05). C) Caspase-3/7 activity (n = 4) measured 24 hours following a 120 minute exposure to varying concentrations of DCA in the presence or absence of 20 uM EIPA (*p<0.05 compared to control). D) MTS assay (n = 16) analyzed 24 hours following a 120 minute exposure to DCA at varying concentrations in the presence or absence of 20 uM EIPA (*p<0.01 compared to control).

Contrast microscopy showed the morphological features of apoptosis, such as cell shrinkage, evident after 2 hours of DCA exposure (Figure 4A yellow arrows). EIPA fully prevented these changes (Figure 4A).

Next, we analyzed cells by Geimsa staining which allows for the visualization and quantification of necrotic and apoptotic cells. The NHE inhibitor EIPA made the cells resistant to DCA-induced apoptosis and to a lesser degree, necrosis (Figure 4B, p<0.001).

To examine the effects of NHE inhibition on DCA-induced apoptosis in a more quantitative manner we measured caspase3/7 activity. Incubation with DCA for 2 hours resulted in a nearly 600% increase in caspase3/7 activity (Figure 4C). The critically important observation was that when NHE was inhibited by EIPA treatment, cells were completely resistant to DCA-induced apoptosis even if at DCA concentrations as high as 0.5 mM DCA (Figure 4C). The inhibition of DCA-induced apoptosis by EIPA was also observed in a different esophageal cell line, CP-A cells (Figure S2).

We used MTS assays as a general method for the evaluation of cell viability and cytotoxicity. Inhibition of NHE by EIPA caused resistance to the cytotoxic effects of DCA (Figure 4D). After 24 hours in the presence of 0.5 mM DCA only 8.6%±2.0% viable cells were observed compared with 95.8%±2.4% viable cells when NHE was inhibited with EIPA (Figure 4D). Importantly, a different NHE inhibitor, dimethylamiloride, had a similar effect (data not shown). Zoniporide, an NHE inhibitor selective for the NHE1 isoform, had a smaller effect than EIPA on DCA-induced cytotoxicity (Figure S2). These results suggest that inhibition of multiple NHE isoforms may be required to fully prevent cell death induced by DCA. Therefore, we examined the expression of NHE isoforms by RT-PCR. CP-A cells were found to primarily express SLC9A1 (NHE1) along with low levels of SLC9A2 (NHE2) and SLC9A3 (NHE3). JH-EsoAd1 cells were found to express SLC9A1 and SLC9A2 and very low levels of SLC9A3 (Figure S3).

### Protein kinase C (PKC) is not involved in DCA-induced NHE activation

PKC is considered crucial for NHE activation [40]. Since DCA has previously been shown to activate PKC [9], [41], we hypothesized that if PKC is responsible for NHE activation then PKC inhibition will prevent changes in the intracellular ion concentrations. JH-EsoAd1 cells were pretreated cells with 10 uM Go6983 for 30 minutes and then exposed to 0.4 mM DCA for 1 hour in the continued presence of 10 uM Go6983. Go6983 is a potent, cell-permeable inhibitor of PKC that inhibits several PKC isozymes (PKC_α_, PKC_β_; PKC_γ_; PKC_δ_; PKC_ζ_). The results indicate Go6983 does not prevent changes in intracellular Na^+^ or K^+^ (Figure S4). On this basis, we concluded that DCA induced changes in intracellular Na^+^ and K^+^ are not affected by PKC.

### DCA induces loss of ATP

ATP levels were measured in JH-EsoAD1 cells after 2 hours exposure to various concentration of DCA in the presence or absence of EIPA. It was observed that DCA at 0.4 mM or 0.5 mM induces significant loss of ATP (p<0.05). EIPA prevented DCA-induced ATP depletion (Figure S6).

### Ouabain and Amphotericin B induce apoptosis in esophageal cells

**Figure 5:**
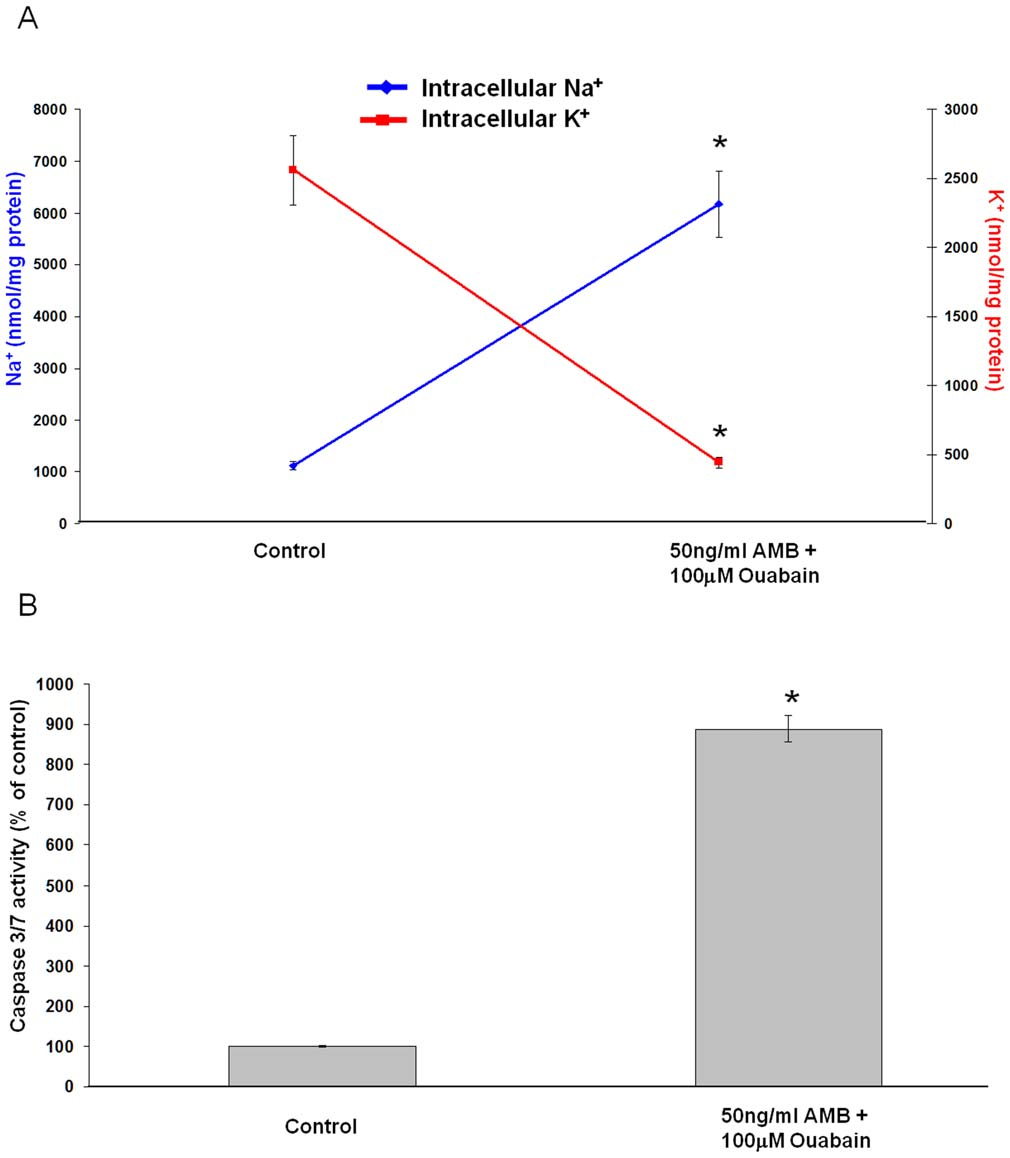
The pharmacologically induced increase of intracellular Na^+^ activates caspase 3/7. A) Total cell Na^+^ (blue trace) and K^+^ (red trace) levels in JH-EsoAd1 cells following incubation with 50 ng/ml amphotericin B (AMB) and 100 uM ouabain for 60 minutes (n = 6). The data are expressed as nmol ion/mg protein (*p<0.001 compared to control). B) Caspase-3/7 activity measured in JHEsoAD1 cells 24 hours following a 120 minute exposure to 50 ng/ml amphotericin B and 100 uM ouabain (*p<0.001 compared to control).

Since a rise of cytoplasmic Na^+^ appeared to be linked to DCA-induced apoptosis, we examined apoptosis in cells in which Na^+^ was increased by exposure to a combination of amphotericin-B (AMB) and ouabain. AMB is a pseudoionophore that stimulates Na^+^ influx, while ouabain, a Na^+^, K^+^ -ATPase inhibitor, prevents active Na^+^ export. In combination AMB (50 ng/ml) and ouabain (100 uM) caused similar changes of intracellular Na^+^ and K^+^ levels to DCA treatment. Importantly, the rise of cytoplasmic Na^+^ and loss of K^+^ (Figure 5A) in cells treated with AMB+ouabain was paralleled by increased caspase-3/7 activation (Figure 5B).

### Low sodium medium inhibits DCA-induced cell death and caspase3/7 activation

**Figure 6:**
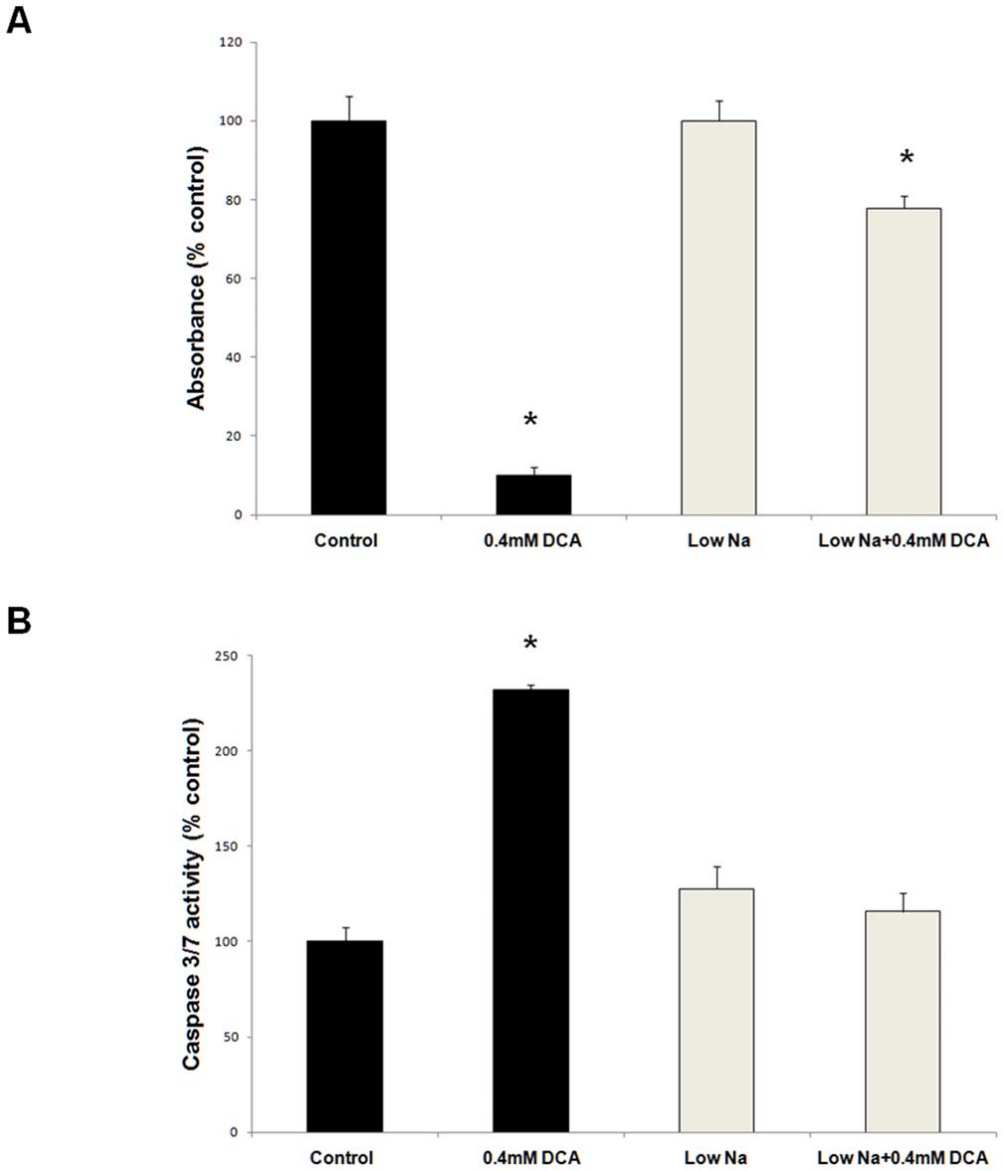
Low sodium medium inhibits DCA-induced cytotoxicity and caspase 3/7 activation. JH-EsoAd1 cells were treated with DCA for 2 hours in Krebs solution with normal Na^+^ (137 mM) or low Na^+^ concentration (20 mM). Panel A shows data from MTS assay and panel B shows caspase 3/7 activation assay performed 24 hours later (*p<0.05 compared to control).

To confirm that the observed changes in intracellular Na^+^ are crucial for DCA-induced apoptosis, JH-EsoAd1 cells were exposed to 0.4 mM DCA in Krebs solution with either normal Na^+^ (137 mM) or reduced Na^+^ concentration (20 mM). After 2 hours the cells were washed and incubated for an additional 24 hours in normal or low sodium Krebs solution. Cytotoxicity was measured by MTS assay and caspase3/7 activity was determined by Apo-ONE® caspase assay. DCA-induced cell death and caspase 3/7 activation was observed in the cells incubated in Krebs solution with 137 mM Na^+^ (Figure 6A,B). However, the cytotoxic effects of DCA and caspase 3/7 activation were significantly reduced when the cells were exposed to DCA in low sodium medium (p<0.05, Figure 6A.B).

### NHE inhibition prevents DCA-induced damage and apoptosis in rat ileum

The above studies with JH-EsoAd1 cells suggest that inhibition of NHE causes apoptosis resistance in DCA-treated cells. Since JH-EsoAd1 cells are derived from a columnar epithelium, we conducted experiments to test whether the response is similar in intact rat ileum. Freshly harvested tissues were treated ex-vivo with control medium or medium with DCA and/or EIPA for 2 hours. The tissues were evaluated for morphological changes by H&E staining and for caspase-3 activation by immunohistochemistry and immunoblotting.

**Figure 7:**
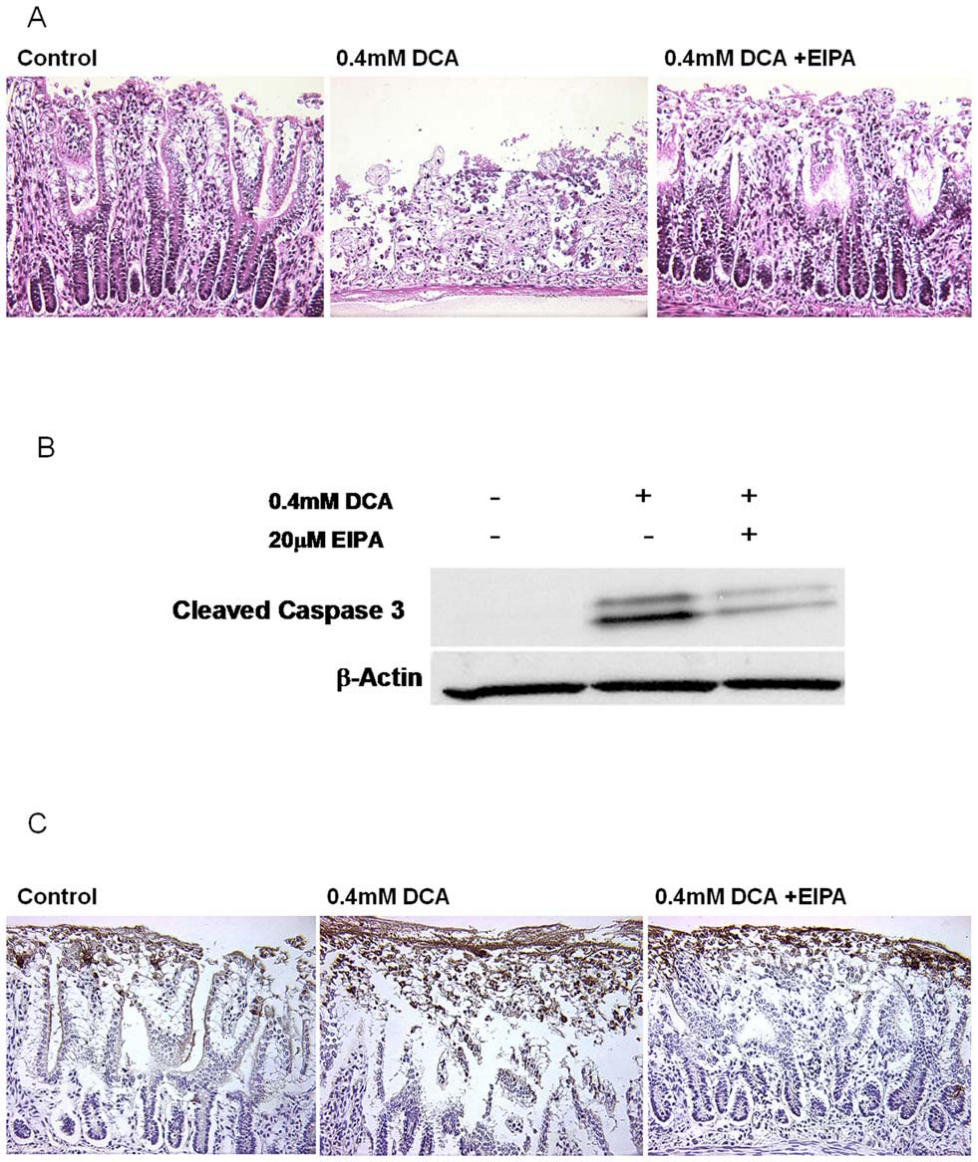
Inhibition of NHE with EIPA prevents DCA-mediated tissue damage and induction of apoptosis in rat ileal epithelium. A) Representative H&E staining of rat ileum treated for 2 hours with or without 0.4 mM DCA in the presence or absence of 20 uM EIPA. B) Western blot of active (cleaved) caspase-3 from homogenized tissue treated for 2 hours with or without 0.4 mM DCA in the presence or absence of 20 uM EIPA. Panel C shows representative immunohistochemical staining of active (cleaved) caspase-3 following the same experimental treatment. The brown color brown indicates active caspase-3.

DCA treatment caused marked disruption of crypts and villi (Figure 7A). However, when EIPA was used to inhibit NHE activity the damaging effects of DCA in tissues were almost fully eliminated (Figure 7A). Western blot analysis of ileum samples revealed an increase in caspase 3 activity induced by DCA treatment. The change in caspase 3 activity was markedly inhibited by EIPA (Figure 7B). Immunohistochemistry revealed an increased signal for cleaved (active) caspase-3 in the epithelial layer of tissue samples that had been exposed to DCA compared to tissues exposed to DCA in the presence of EIPA (Figure 7C).

## Discussion

Bile acids are important in the etiology of gastrointestinal cancers including EAC [3], [42], [43]. During reflux episodes bile acids along with gastric acids are refluxed in to the esophagus. While acute exposure to bile acids leads to the induction of apoptosis, it has been proposed that chronic, repeated exposures to bile acids may lead to selection of apoptosis-resistant cell clones [44], [45]. In this context, apoptosis is an important protective mechanism for elimination of cells with bile acid-induced DNA damage. Apoptosis resistance is one of the hallmarks of cancer cells. To understand how apoptosis resistance develops, we seek to understand the early steps that activate the apoptotic pathway in response to bile acid exposure. Here, we report a novel mechanism of bile-acid induced apoptosis involving NHE and ionic imbalance. We present data using JH-EsoAd1 esophageal cancer cells. Similar results were observed in another esophageal cell line (CP-A cells) derived from Barrett's esophagus (Figure S1, S5).

We know from the previous studies that bile acids cause cytoplasmic acidification [21]. Lysosomes are the most acidic organelles in the cell [46] and lysosomal damage involving lysosomal membrane perturbation is known to reduce cytoplasmic pH [38], [46]. Changes in the subcellular distribution of Lysotracker-Green and Red, fluorescent dyes which accumulate in acidic lysosomes, were consistent with lysosomal membrane perturbation in DCA-treated cells [37]. Reduced expression of LAMP1 (a lysosomal marker) and electron microscopy confirmed lysosomal damage. The NHE inhibitor EIPA had no influence on the extent of lysosomal damage in DCA-treated cells. Taken together, these results suggest that DCA induces lysosomal membrane perturbation that reduce cytoplasmic pH while NHE activation and Na^+^ influx come later in the chain of events, in response to cytoplasmic acidification. According to this scheme it is understandable that lysosomal damage could not be rescued by the addition of EIPA.

We found that DCA induces an increase in intracellular Na^+^ in a dose-dependent manner in esophageal cells. The NHE inhibitor EIPA prevents the Na^+^ increase. We interpret the results as an indication that NHE is the transporter responsible for Na^+^ influx in DCA-treated cells. DCA caused a transient acidification but after 20 minutes cytoplasmic pH returned to normal. In contrast, DCA caused large and long lasting pH reduction in cells exposed to EIPA. The observations are consistent with the notion that NHE is activated in order to export H^+^ and to stabilize intracellular pH in DCA-treated cells. Because NHE exchanges H^+^ for Na^+^, NHE activation by DCA increases intracellular Na^+^.

K^+^ is lost from the cells to stabilize osmolarity when cellular Na^+^ increases [23], [24]. JH-EsoAd1 cells showed a significant decrease of intracellular K^+^ levels in the presence of DCA (Figure 3A,B). Inhibition of NHE with EIPA prevented the DCA-induced loss of K^+^ (Figure 3A). EIPA also abolished the DCA-induced rise of cytoplasmic Ca ^2+^.

Na^+^ increase and K^+^ loss [47] are commonly associated with a rise of cytoplasmic Ca^2+^ in part because the driving force for Na^+^-Ca^2+^ transport is reduced. Ca^2+^ is vital to different cellular signaling processes including protein kinase activation. Critically, a calcium rise can trigger apoptosis [47], [48].

Gerbino et al. speculated that efflux of K^+^ and influx of Ca^2+^ through Ca^2+^-activated K^+^ channels is responsible for the initiation of apoptosis by DCA. However, pharmacologic inhibition of Ca^2+^-activated K^+^ channels with the combination of two drugs, apamin and charybdotoxin, decreased DCA-induced apoptosis only by ∼50% [16]. Our studies suggest a different mechanism. We propose that Na^+^ influx is mediated by NHE, as a consequence of acidification. It is the initiating event for both K^+^ loss and Ca^2+^ increase. This chain of events has been recognized previously by Bortner and co-workers who demonstrated that Na^+^ influx is required for ionic imbalance and apoptosis in lymphoma tumor cells implicating an unknown plasma membrane transporter [25].

Importantly, the present results clearly show that cells can tolerate acidification and still evade DCA-induced apoptosis if NHE is inhibited by EIPA, zoniporide or DMA or if low sodium medium is used to prevent sodium entry. Although several studies suggest intracellular acidification is important for induction of apoptosis [49], [50], [51], the current studies imply that ion imbalance induced by NHE activation is more important than cellular acidification. In the presence of EIPA, DCA induced severe cellular acidification, but caspase 3/7 activity remained low. In contrast, after 60 minutes treatment with DCA alone, the reduction of cytoplasmic pH was small and transient but intracellular Na^+^, Ca^2+^, caspase activity and apoptosis, all displayed a large increase (Figures 1B). The findings suggest that the cytoplasmic Na^+^ increase caused by NHE activation is a key initiating factor for apoptosis induction and not intracellular acidification. This notion was reinforced by the observation that caspase activation could be triggered by a rise of cytoplasmic Na^+^ independent of DCA treatment.

EIPA has been previously implicated in preventing cell death [52], [53]. This the first report showing that bile acids-induced apoptosis is critically dependent on NHE activation and that treatment with EIPA prevents apoptosis. Nevertheless, ion-mediated cell death is important but it is unlikely to be the only mechanism of bile acid-induced apoptosis. Other mechanisms such as CD95-mediated apoptosis could be responsible for the low levels of apoptosis observed in the cells treated with high concentrations of DCA (0.5 mM) in the presence of EIPA.

Intracellular Na^+^ levels are tightly controlled by Na^+^,K^+^-ATPase, which is expressed ubiquitously. Na^+^,K^+^-ATPase acts as an ion pump. In each cycle it moves three Na^+^ ions out of cell and two K^+^ ions move inward, using energy from ATP hydrolysis. We found that ATP levels are significantly decreased after DCA exposure (Figure S6). Lack of ATP could explain why the intracellular Na^+^ is not normalized by Na^+^,K^+^-ATPase. In the presence of EIPA the DCA-induced reduction in ATP was not observed, likely because Na^+^ entry is suppressed, so eliminating the increased demand of Na^+^,K^+^-ATPase for ATP (Figure S6).

Our previous report suggests that BE tissues display resistance to apoptosis in response to DCA [7]. In the current study we show the critical role of NHE in bile acid-induced apoptosis. We hypothesize that after chronic, repeated exposures to bile acids, the cells develop protective mechanisms that partially suppress NHE activity and Na^+^ influx. Endogenous nitric oxide produced by nitric oxide synthase (NOS) was shown to partially inhibit NHE activity [21], [22]. Interestingly, increased NO levels are associated with resistance to apoptosis. For example, Gumpricht et al showed that treatment with the NO donors inhibited bile acid-induced apoptosis and caspase 3 activation by a non-mitochondrial-dependent pathway [54].

Our earlier studies indicate that NOS is immediately activated by bile acids, which leads to NO production and partial inhibition of NHE [21]. We suggest that this is one of the mechanisms that protect cells from apoptosis. It is noteworthy, that inducible NOS was found to be increased in esophageal cancer [55], [56], [57]. It is our next goal to elucidate the role of NO and NHE in apoptosis resistance. We recently developed CP-A cells that are able to grow in the medium containing 0.2 mM DCA and we are planning to evaluate the mechanism of apoptosis protection in these cells.

**Figure 8:**
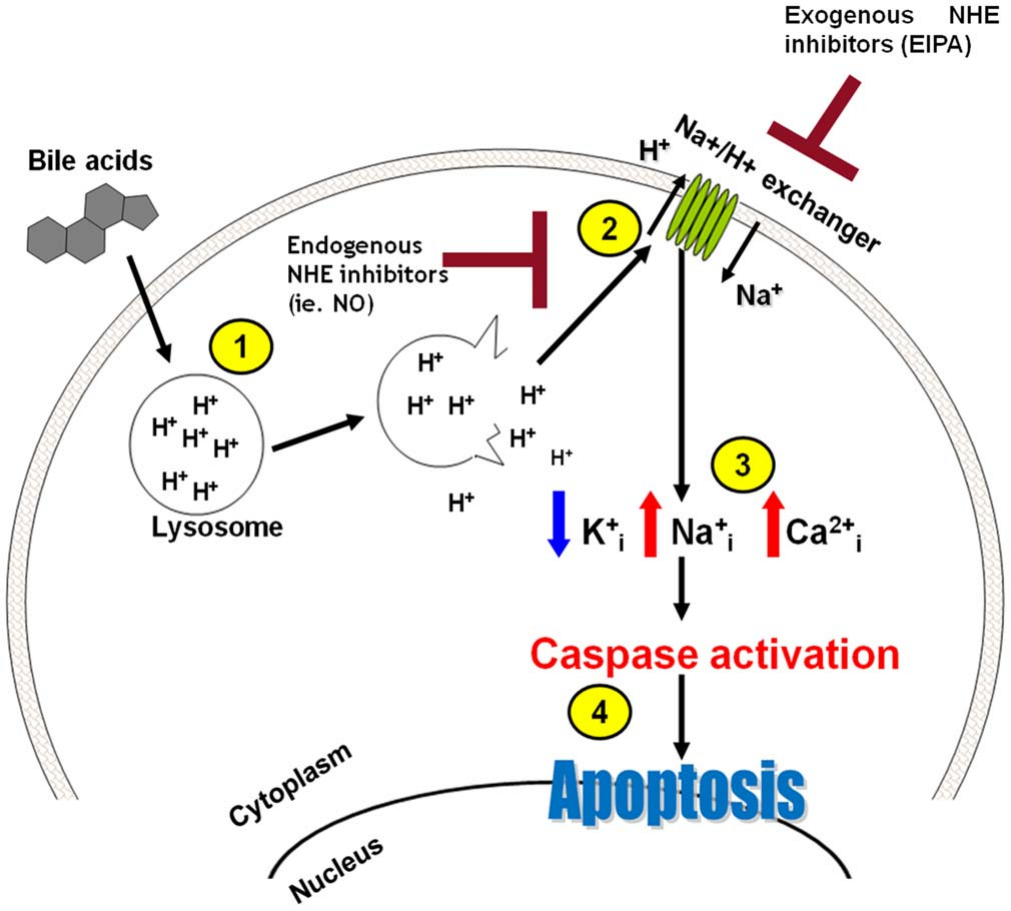
Proposed scheme of DCA-induced apoptosis and the role of NHE inhibitors in apoptosis-resistance. (1) Bile acids damage lysosomes and release the acidic contents into the cytoplasm. (2) Cytoplasmic acidification leads to NHE activation. (3) Na^+^ enters cells in exchange for H^+^. As a result K^+^ exits cells to balance osmolarity and cytoplasmic calcium rises because Na^+^-Ca^2+^ exchange is impaired. (4) Ion imbalance leads to caspase activation and apoptosis. NO or other endogenous inhibitors inhibit NHE and tend to prevent apoptosis induction.

The current study shows that NHE activation and the accompanying intracellular Na^+^ increase are critical steps leading to bile acid-induced apoptosis. The cells evade DCA-induced apoptosis when NHE is inhibited by EIPA even though the intracellular pH decreases dramatically. In addition to NO, several other endogenous signals have been shown to suppress NHE activity in gastrointestinal cells [17]. Many of these signals are linked to gastrointestinal tumorigenesis [17]. The scheme in Figure 8 presents our proposed mechanism of DCA-induced apoptosis as a function of ionic dysbalance. We suggest that the endogenous inhibitors of NHE may contribute to apoptosis resistance.

## Supporting Information

Figure S1 **Lysosomal damage accompanies DCA-mediated intracellular acidification.** A) Representative fluorescent microscopy images showing Lysotracker-Red in CP-A cells following 60 minutes treatment with or without 0.4 mM DCA in the presence or absence of 20 uM EIPA. Red signal indicates acidic vesicles, blue signal is nuclear counterstain (DAPI). B) Representative electron microscopy images (25,000×) of cells treated with or without 0.4 mM DCA in the presence or absence of 20 uM EIPA. Red arrows indicate structurally intact lysosomes, yellow arrows indicate lysosomal membrane perturbation. C) Representative confocal microscopy images showing LAMP-1 (green signal) and nuclear stain for propidium iodide (red signal) in CP-A cells subjected the same treatments.
(TIF)

Figure S2 **Zoniporide prevents DCA-induced cell death.** The graph shows data from MTS assay (n = 4) in JHEsoAd1 cells detected 24 hours following a 120 minute exposure to 0.4 mM DCA in the presence or absence of 20 mM zoniporide (*p<0.05).
(TIF)

Figure S3 **NHE1, NHE2 and NHE3 mRNA detected in CP-A cells and JHEsoAD1 cells.** mRNA levels were measured by RT-PCR from mRNA obtained from three independent experiments (*p<0.05 compared to CP-A cells).
(TIF)

Figure S4 **PKC inhibition does not prevent changes in intracellular Na^+^ and K^+^. in JHEsoAd1 cells treated with DCA.** JHEsoAd 1 cells were pretreated for 30 minutes with 10 mM Go6983 and then exposed to 0.4 mM DCA for 60 minutes in the presence or absence of Go6983 (n = 3; *p<0.05 compared to control).
(TIF)

Figure S5 **Inhibition of Na^+^ influx with EIPA prevents DCA-induced cell death in CP-A cells.** A) Representative contrast microscopy images of CP-A cells following 120 minute incubation with and without 0.4 mM DCA in the presence or absence of 20 uM EIPA. Yellow arrows indicate damaged and apoptotic cells. B) Caspase-3/7 activity (n = 4) measured 24 hours following a 120 minute exposure to varying concentrations of DCA in the presence or absence of 20 uM EIPA (*p<0.05 compared to control).
(TIF)

Figure S6 **DCA induced ATP depletion in JHEsoAD1 cells.** The cells were exposed for 2 hours to various concentration of DCA in the presence or absence of EIPA and ATP levels were measured by Enliten ATP Assay System Bioluminiscence Kit according the manufacturer's instructions. EIPA prevents ATP depletion (*p<0.05 compared to control).
(TIF)
