# Supplementary figures and images for: The Na+/H+ Exchanger Controls Deoxycholic Acid-Induced Apoptosis by a H+-Activated, Na+-Dependent Ionic Shift in Esophageal Cells
Source: PLoS One. 2011 Aug 22;6(8):e23835. doi: 10.1371/journal.pone.0023835 (PMC3161789; doi:10.1371/journal.pone.0023835)

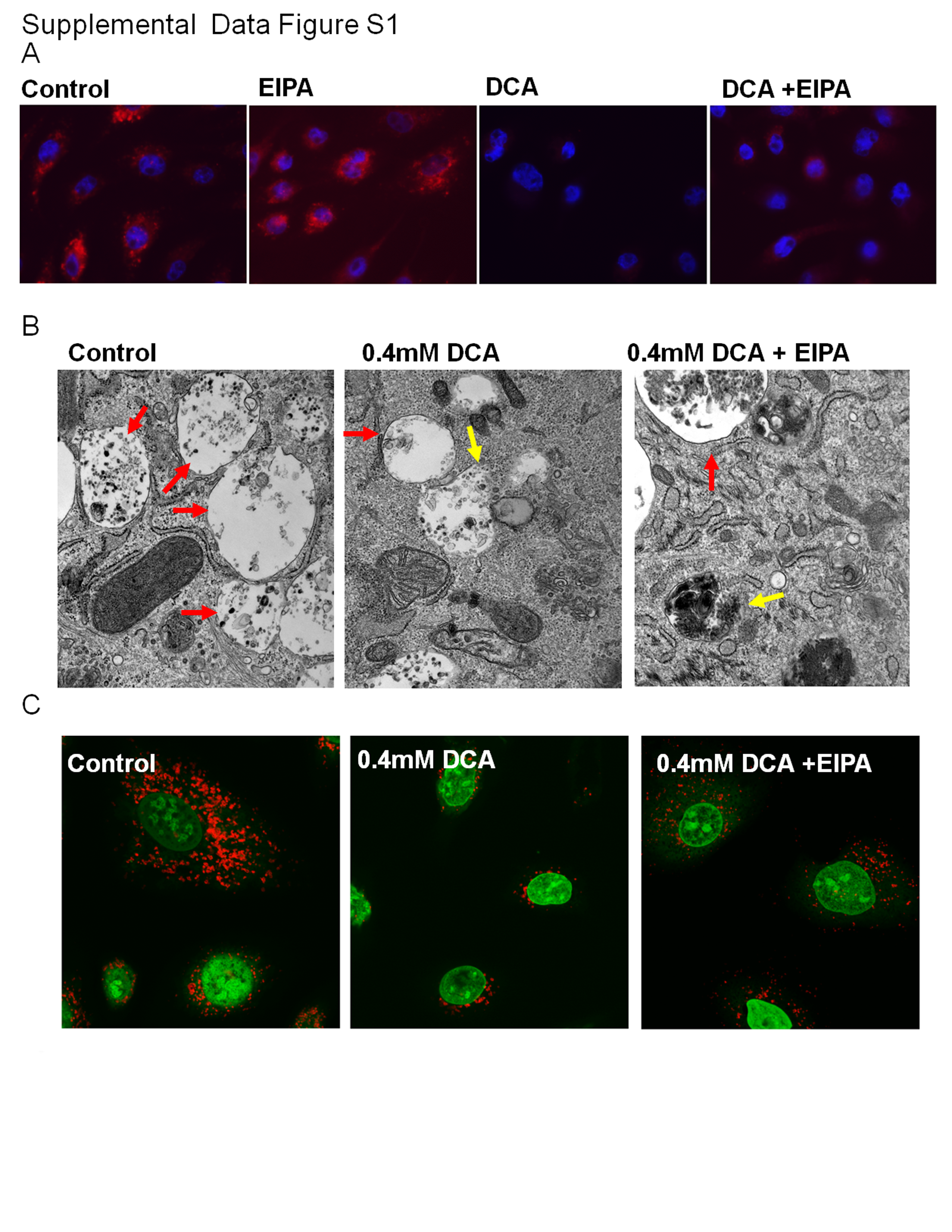

Supplement: Figure S1 — Lysosomal damage accompanies DCA-mediated intracellular acidification. A) Representative fluorescent microscopy images showing Lysotracker-Red in CP-A cells following 60 minutes treatment with or without 0.4 mM DCA in the presence or absence of 20 uM EIPA. Red signal indicates acidic vesicles, blue signal is nuclear counterstain (DAPI). B) Representative electron microscopy images (25,000×) of cells treated with or without 0.4 mM DCA in the presence or absence of 20 uM EIPA. Red arrows indicate structurally intact lysosomes, yellow arrows indicate lysosomal membrane perturbation. C) Representative confocal microscopy images showing LAMP-1 (green signal) and nuclear stain for propidium iodide (red signal) in CP-A cells subjected the same treatments. (TIF) [file pone.0023835.s001.tif]

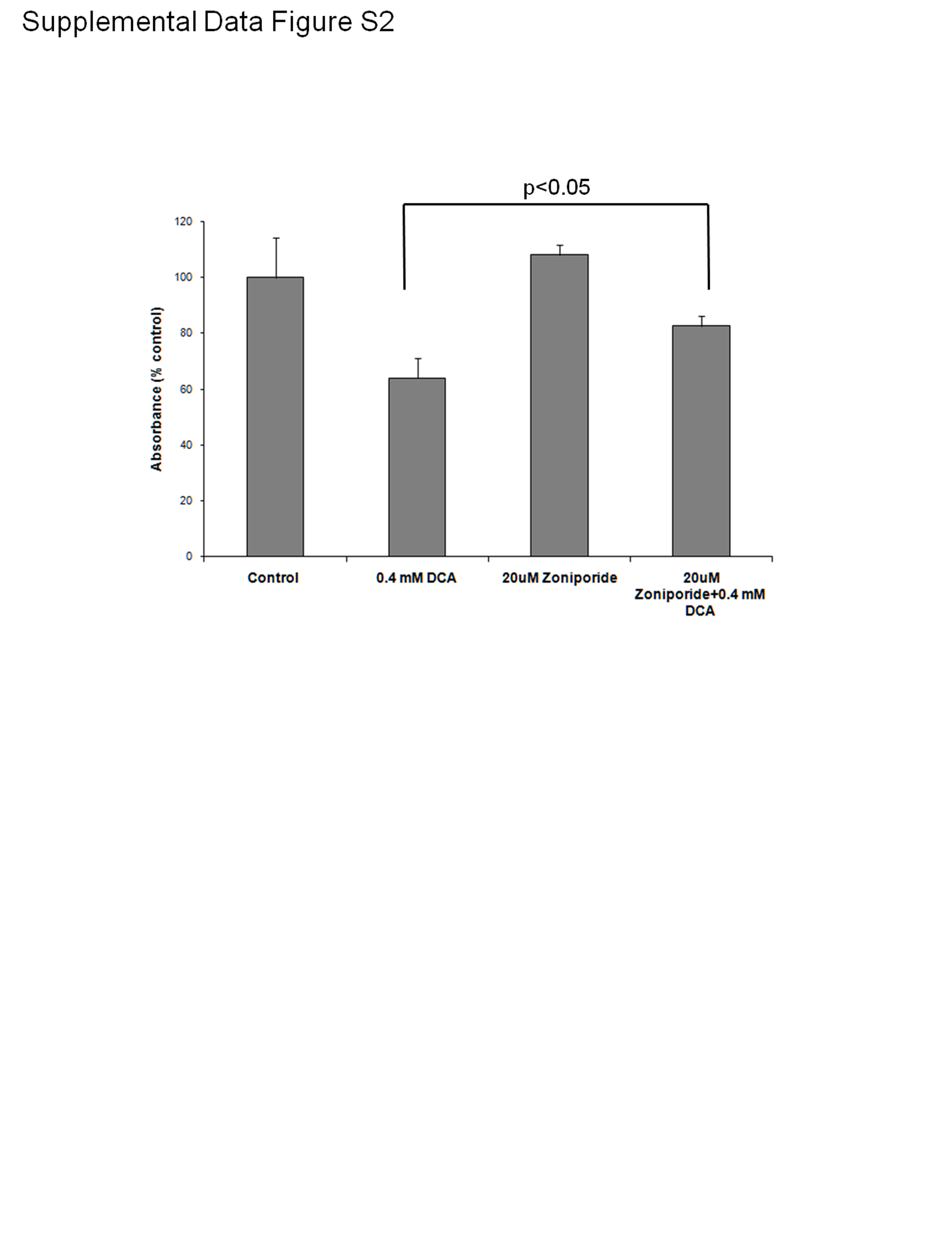

Supplement: Figure S2 — Zoniporide prevents DCA-induced cell death. The graph shows data from MTS assay (n = 4) in JHEsoAd1 cells detected 24 hours following a 120 minute exposure to 0.4 mM DCA in the presence or absence of 20 mM zoniporide (*p<0.05). (TIF) [file pone.0023835.s002.tif]

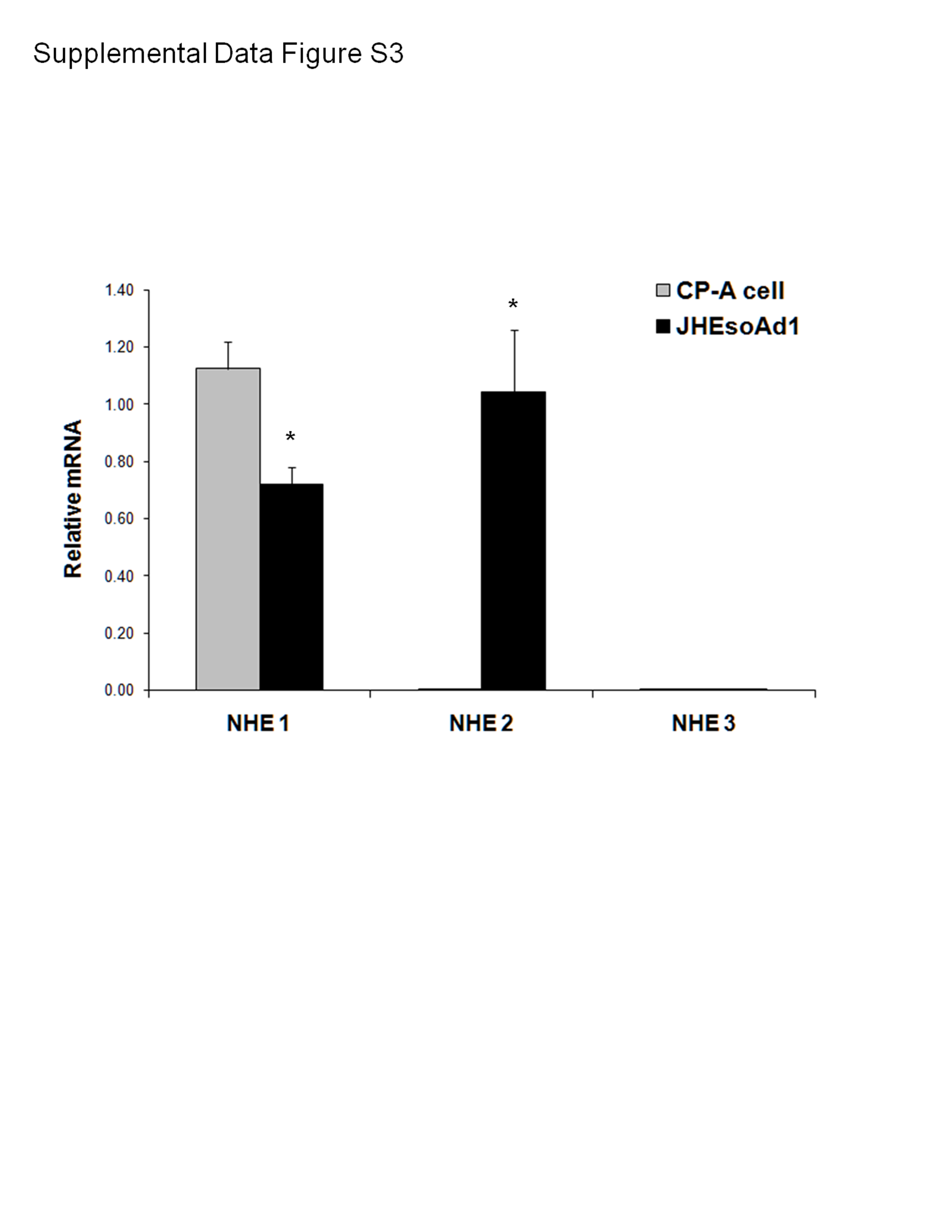

Supplement: Figure S3 — NHE1, NHE2 and NHE3 mRNA detected in CP-A cells and JHEsoAD1 cells. mRNA levels were measured by RT-PCR from mRNA obtained from three independent experiments (*p<0.05 compared to CP-A cells). (TIF) [file pone.0023835.s003.tif]

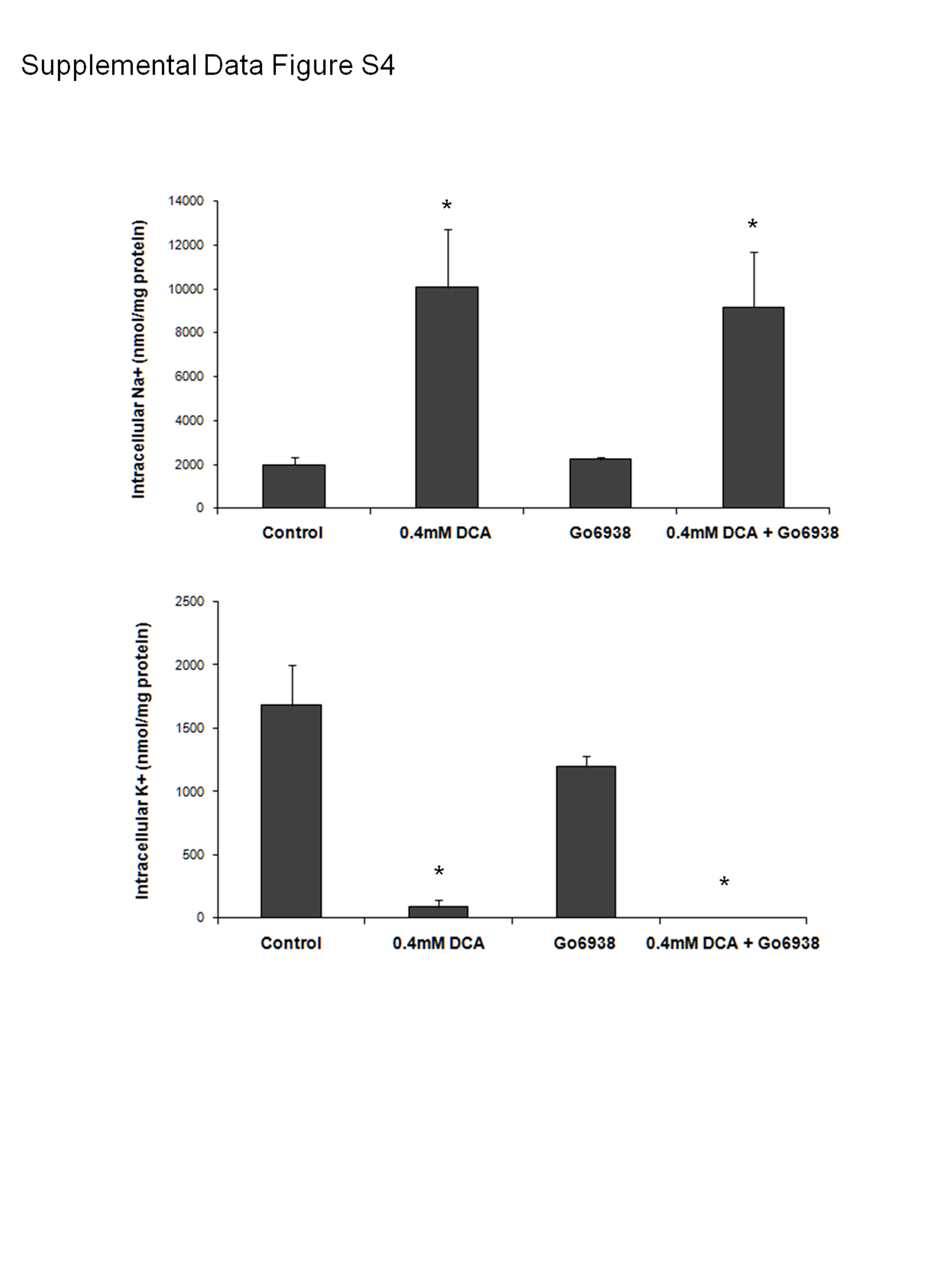

Supplement: Figure S4 — PKC inhibition does not prevent changes in intracellular Na+ and K+. in JHEsoAd1 cells treated with DCA. JHEsoAd 1 cells were pretreated for 30 minutes with 10 mM Go6983 and then exposed to 0.4 mM DCA for 60 minutes in the presence or absence of Go6983 (n = 3; *p<0.05 compared to control). (TIF) [file pone.0023835.s004.tif]

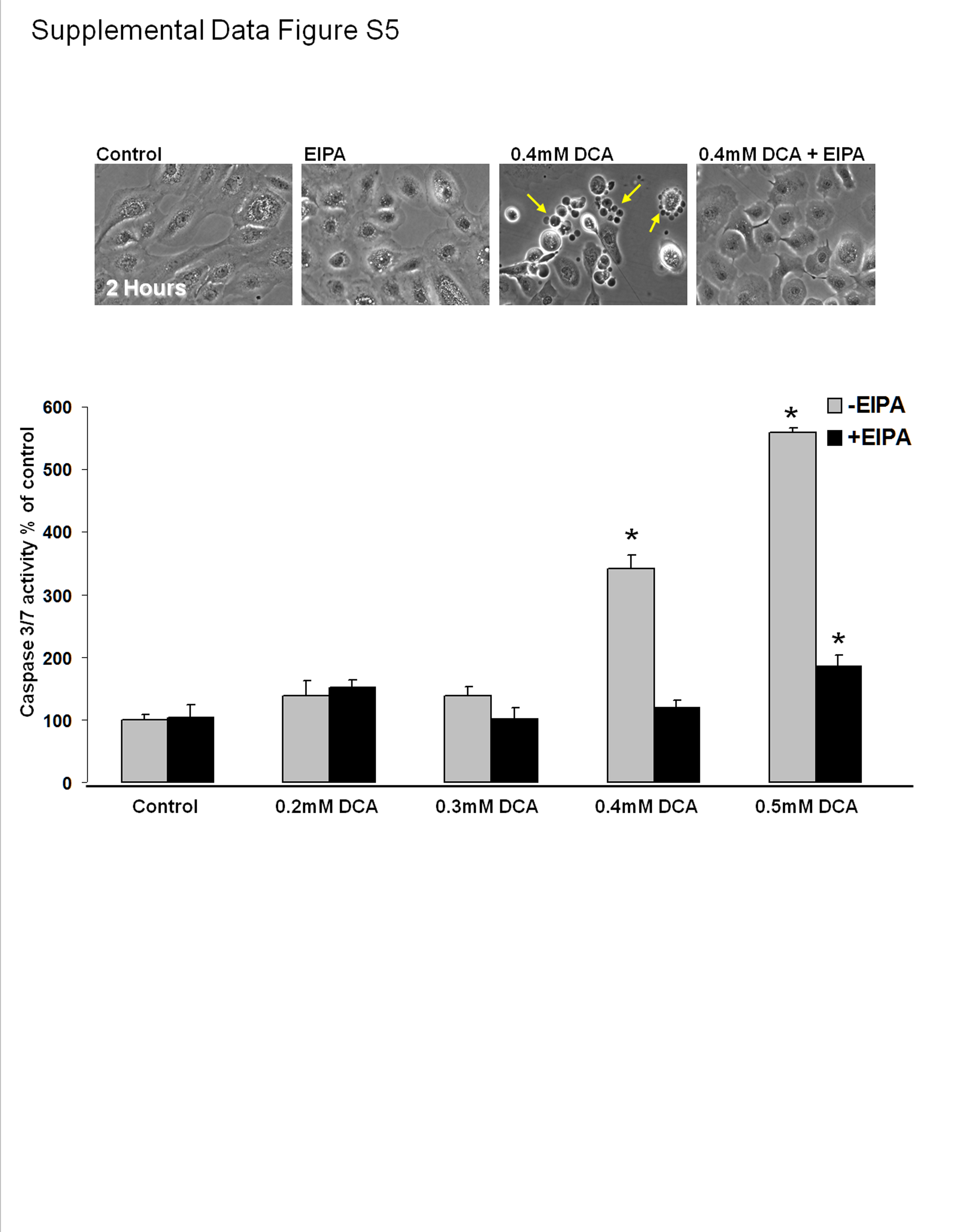

Supplement: Figure S5 — Inhibition of Na+ influx with EIPA prevents DCA-induced cell death in CP-A cells. A) Representative contrast microscopy images of CP-A cells following 120 minute incubation with and without 0.4 mM DCA in the presence or absence of 20 uM EIPA. Yellow arrows indicate damaged and apoptotic cells. B) Caspase-3/7 activity (n = 4) measured 24 hours following a 120 minute exposure to varying concentrations of DCA in the presence or absence of 20 uM EIPA (*p<0.05 compared to control). (TIF) [file pone.0023835.s005.tif]

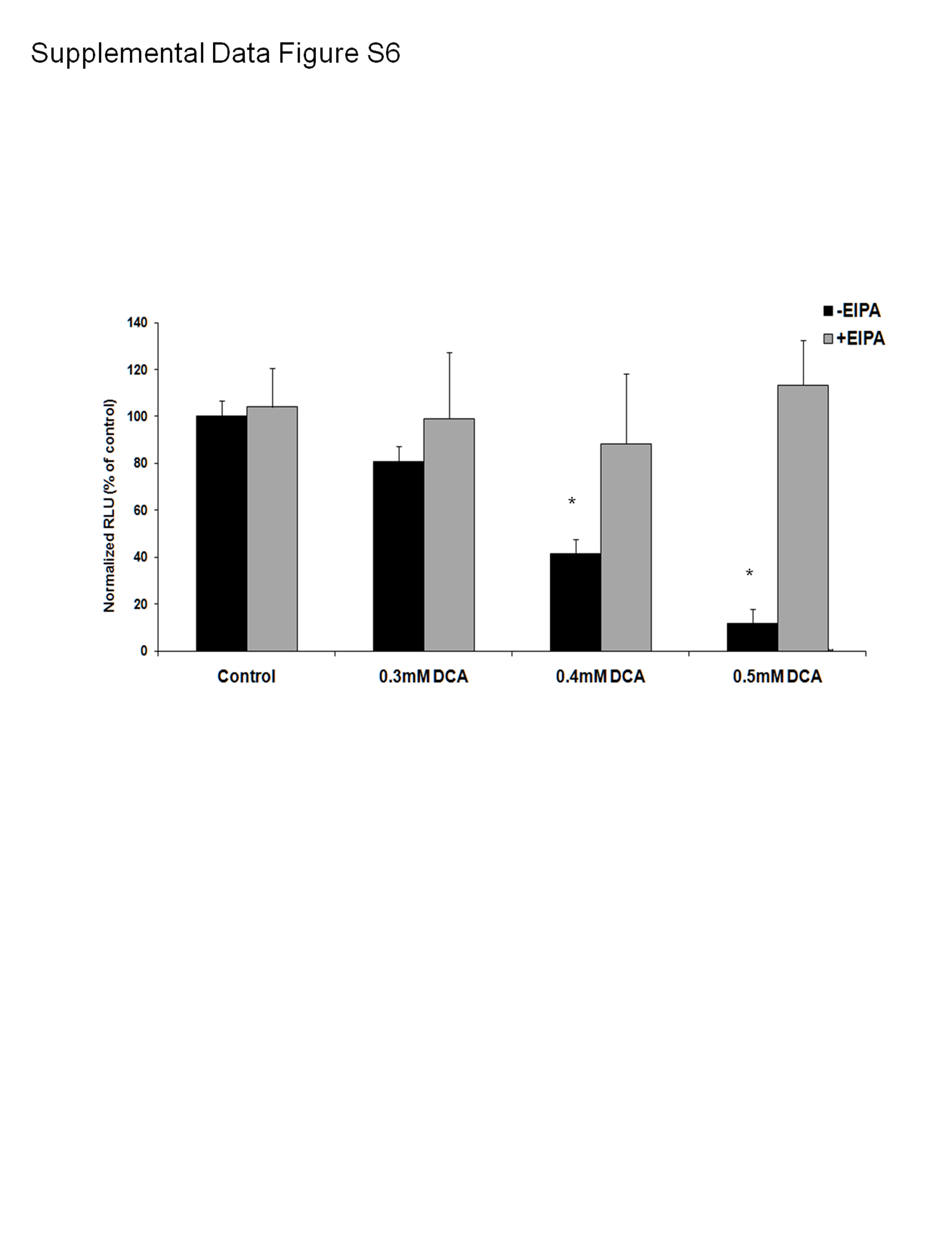

Supplement: Figure S6 — DCA induced ATP depletion in JHEsoAD1 cells. The cells were exposed for 2 hours to various concentration of DCA in the presence or absence of EIPA and ATP levels were measured by Enliten ATP Assay System Bioluminiscence Kit according the manufacturer's instructions. EIPA prevents ATP depletion (*p<0.05 compared to control). (TIF) [file pone.0023835.s006.tif]
